# Factor H-Related (FHR)-1 and FHR-2 Form Homo- and Heterodimers, while FHR-5 Circulates Only As Homodimer in Human Plasma

**DOI:** 10.3389/fimmu.2017.01328

**Published:** 2017-10-18

**Authors:** Anna E. van Beek, Richard B. Pouw, Mieke C. Brouwer, Gerard van Mierlo, Judy Geissler, Pleuni Ooijevaar-de Heer, Martin de Boer, Karin van Leeuwen, Theo Rispens, Diana Wouters, Taco W. Kuijpers

**Affiliations:** ^1^Department of Immunopathology, Sanquin Research and Landsteiner Laboratory of the Academic Medical Centre, University of Amsterdam, Amsterdam, Netherlands; ^2^Department of Pediatric Hematology, Immunology and Infectious Diseases, Emma Children’s Hospital, Academic Medical Centre, Amsterdam, Netherlands; ^3^Department of Blood Cell Research, Sanquin Research and Landsteiner Laboratory of the Academic Medical Centre, University of Amsterdam, Amsterdam, Netherlands

**Keywords:** innate immunity, complement factor H, factor H-related proteins, CFHR1, CFHR2, CFHR5, dimerization, quantification

## Abstract

The complement factor H-related (FHR) proteins are hypothesized to fine-tune the regulatory role of complement factor H (FH) in the alternative pathway of the complement system. Moreover, FHR-1, FHR-2, and FHR-5 have been proposed to be dimers, which further complicates accurate analysis. As FHRs are highly similar among themselves and toward FH, obtaining specific reagents for quantification of serum levels and functional analysis is challenging. In this study, we generated antibodies and developed ELISAs to measure FHR-1, FHR-2, and FHR-5 in serum. We used both recombinant and serum-derived proteins to show that four dimers occur in human circulation: homodimers of FHR-1, FHR-2, and FHR-5, as well as FHR-1/FHR-2 heterodimers. Heterodimers containing FHR-5 were not found. In individuals with homozygous *CFHR1* deletions or compound heterozygous *CFHR2* missense/nonsense mutations identified in this study, the respective FHR-1 and FHR-2 homo- and heterodimers were absent. Using FRET, we found that recombinant FHR dimers exchange monomers rapidly. This was confirmed *ex vivo*, using FHR-1- and FHR-2-deficient sera. Of all FHR dimers, FHR-5/5 homodimers demonstrated strong binding affinity toward heparin. Specific ELISAs demonstrated that serum levels of FHR-1/1, FHR-1/2, FHR-2/2, and FHR-5/5 dimers were low compared to FH, which circulates at a 10- to 200-fold molar excess. In summary, FHR-1, FHR-2, and FHR-5 homodimerize, with FHR-1 and FHR-2 forming heterodimers as well, and equilibrate quickly in plasma.

## Introduction

Protection of human cells against unwanted complement activation is achieved by complement factor H (FH), the major regulator of the alternative pathway. FH is an abundant plasma glycoprotein that circulates at an average concentration of approximately 300 µg/mL and consists of 20 short consensus repeat domains (SCRs) (1–4). While SCR1–4 are involved in the regulatory activity of FH as a cofactor for factor I and facilitate the decay of the C3 convertase, SCR6–7 and SCR19–20 mediate binding toward C3b and glycosaminoglycans on cellular surfaces (5, 6). Genetic variations in the surface-binding domains of FH are associated with complement-mediated diseases, including age-related macular degeneration (AMD) and atypical hemolytic uremic syndrome (aHUS) (7, 8).

Factor H is part of the FH protein family, also including the *CFH* splice variant, FH-like-1, and the FH-related (FHR) proteins, named FHR-1, FHR-2, FHR-3, FHR-4A, FHR-4B, and FHR-5, each of which is encoded by their own gene, with FHR-4A and FHR-4B being splice variants of *CFHR4*. Limited data are available on the physiological function of the FHRs, although copy number variations (CNV) (9, 10), internal duplications (11–13), fusion proteins (14–18), and polymorphisms (19–22) have been described that are associated with complement-mediated diseases. While most of their sequence similarity is towards the surface-binding domains of FH, none of the FHRs seem to possess domains similar to the regulatory domains of FH. This led to the hypothesis that FHRs compete with FH for C3b and surface binding without regulating C3b, and thereby fine-tune the inhibitory role of FH in complement activation on host and pathogenic cellular surfaces.

Factor H-related-1, FHR-2, and FHR-5 contain a dimerization motif in their first two SCR domains, proposed to result in homo- and heterodimerization (23). A crystal structure of recombinant FHR-1^SCR1/SCR2^ revealed a head-to-tail dimer composition of the first two domains with SCR1 on the one protein interacting with SCR2 of the other protein. This also revealed the presence of three critical residues that stabilize dimerization and are present in FHR-1, FHR-2, and FHR-5, suggesting that the three proteins would homo- and heterodimerize with each other. Dimerization was shown to increase the avidity of FHR-1 and FHR-5 for C3b and enhance complement activation, by enhancing their competition with FH activity in a guinea pig erythrocyte hemolysis assay (11, 23, 24). Although homodimerization has been confirmed for recombinant (r) FHR-1 and rFHR-2 (24, 25), the presence of dimers in plasma has not yet been formally demonstrated.

Due to the high sequence similarity between FH and the FHRs, it is a major challenge to obtain highly specific tools that allow for detailed characterization of FHR dimers *in vivo*. We and others have succeeded in accurate and specific measurements of serum levels of FHR-3 and FHR-4 (26–28), while others have been able to measure FHR-5 and FHR-1, although possible homo- and heterodimerization was not taken into account in the measurements (29–31). Some have only succeeded in determining relative levels of FHR-1 (32), possibly affected by cross-reactivity of the reagents used, while none, to our knowledge, have been able to measure FHR-2.

Sequence similarity between FHRs divides these proteins into two groups: one consisting of FHR-3 and FHR-4, the other of FHR-1, FHR-2, and FHR-5. Since the latter three FH-related proteins were suggested to dimerize, we further investigated the possible impact of dimerization on their function, as well as the composition and prevalence of the FHR dimers *in vivo*. We demonstrated that most, but not all, suggested FHR homo- and heterodimers are present in serum, and we further elucidated the kinetics of this dimerization using a Förster resonance energy transfer (FRET)-based assay. Monomers were not identified in serum. We showed that the binding strength toward heparin is affected by the dimer composition and determined the accurate levels of each dimer.

## Materials and Methods

### Samples

Blood samples were obtained from anonymous, healthy volunteers with informed, written consent in accordance with Dutch regulations, and this study was approved by the Sanquin Ethical Advisory Board in accordance with the Declaration of Helsinki. The previously described cohort was extended with new donors of which serum and EDTA plasma were collected and analyzed as reported (26). DNA was extracted from peripheral blood leukocytes to determine CNV by multiplex ligation-dependent probe amplification (MLPA; MRC Holland, Amsterdam, the Netherlands) (26). Pooled serum of >400 healthy donors was a gift from Sanquin Diagnostic Services (Sanquin, Amsterdam, the Netherlands) and used as a standard curve in all ELISAs except the FHR-2/2 homodimer ELISA, in which pooled serum of four donors with a homozygous *CFHR3/CFHR1* deletion was used.

### Proteins and Monoclonal Antibodies

Rat anti-mouse kappa (RM-19) monoclonal antibody (mAb), high-performance ELISA buffer (HPE), and streptavidin conjugated to poly-horseradish-peroxidase (strep-poly-HRP) were purchased from Sanquin Reagents (Amsterdam, the Netherlands); streptavidin conjugated to horseradish-peroxidase (strep-HRP) was obtained from Pierce/Thermofisher Scientific (Waltham, MA, USA). Nanogam [intravenous immunoglobulins (IVIg)] was purchased from Sanquin Plasma Products (Amsterdam, the Netherlands). The monospecific anti-FHR-2 mAb was purchased from R&D Systems (clone MAB5484, Minneapolis, MN, USA); the polyclonal goat anti-human-FH from Quidel (San Diego, CA, USA), conjugated in-house with HRP. mAb anti-FH.02 was generated against FH, in-house as part of another study at our laboratory (directed against SCR20; manuscript in preparation). Proteins were labeled with EZ-link Sulfo-NHS-LC-Biotin, No-Weigh Format according to manufacturer’s instructions (Pierce/Thermofisher Scientific). Sulfo-SMCC and M2 anti-FLAG mAb (F1804) were obtained from Sigma (St. Louis, MO, USA).

### Recombinant Expression of FHRs

Recombinant human FHR (rFHR) proteins, containing a C-terminal 6×-histidine (6×His) tag, were produced and purified as previously described (26). For FHR-1, the CFHR1*A allotype sequence was used (33). In short, proteins were expressed by transient transfection of pcDNA3.1 expression vectors in HEK293F cells, after which proteins were purified from the supernatant by Ni^2+^ affinity chromatography using HisTrap™ High Performance 1 mL columns (GE Healthcare Life Sciences, Freiburg, Germany). rFHRs were filtrated and concentrated using Amicon^®^ Ultra Centrifugal Filter Devices (Merck Millipore, Darmstadt, Germany).

### FRET Assay for Monomer Exchange

Monomer exchange reactions were recorded in real-time with a previously developed FRET assay (34–36). rFHR-2 and rFHR-5 were labeled with DyLight 488 or 594 (Pierce/Thermofisher Scientific) with a labeling degree of 3 to 4 labels per monomer. Experiments were performed at 37°C in quartz cuvettes with an inner chamber of 0.25 mL. Reactions were carried out at 200 ng/mL in degassed PBS in presence of 1 mg/mL IVIg (Nanogam, Sanquin) to prevent adsorption of fluorescent protein to the cuvette wall. To assess monomer exchange between 488- and 594-labeled protein, equimolar amounts were mixed and monitored in time. Monomer exchange between fluorescently labeled and unlabeled protein was carried out by pre-mixing and equilibrating equimolar amounts of 488- and 594-labeled protein, after which a >5 times molar excess of unlabeled protein was added. Fluorophores were excited at 488 nm in a 60 s cycle using a Cary Eclipse fluorescence spectrophotometer (Varian, Mulgrave, VIC, Australia). The appearance of a FRET signal at 620 nm was used to monitor kinetics, while emission at 588 nm was used to account for baseline drifts during reactions (34, 35). Monomer exchange rates were analyzed by fitting a first-order exponential (Graphpad Prism, version 6.04; Graphpad Software, La Jolla, CA, USA).

### Immunization and Hybridoma Generation

Murine mAbs directed against FHR-1, FHR-2, and FHR-5 were generated as previously described (26). The mAbs were screened for cross-reactivity against all rFHRs and plasma-derived FH and were assessed by ELISA to determine their competition for similar binding epitopes. Four mAbs were monospecific for FHR-5, but none were monospecifically detecting FHR-1 or FHR-2. Three mAbs reacted with recombinant protein only: two directed against all FHRs, one directed against rFHR-5.

### Immunoprecipitations and Western Blots

Factor H-related proteins were precipitated from 200 µL human serum using 50 µL of 1 mg/mL anti-FH-02, 100 µg/mL anti-FHR-2, or 100 µg/mL anti-FHR-5.1, as indicated, captured on 500 µL of 5 mg/mL CNBr-activated Sepharose (GE Healthcare), coupled with RM-19 (25 mg mAb per 1 g sepharose), all diluted in PBS supplemented with 0.1% (w/v) Tween-20, 0.1% (w/v) BSA, 10 mM EDTA and incubated rotating o/n at 4°C. Sepharose was washed five times in 1 mL PBS supplemented with 0.1% (w/v) Tween-20 (PT) and eluted with 50 µL 1× NuPAGE LDS Sample Buffer (Invitrogen) before heating at 70°C for 10 min. SDS-PAGE was performed on non-reducing Novex NuPAGE 4–12% Bis–Tris gels (Invitrogen), followed by Western blot onto nitrocellulose membranes (Novex iBlot Gel Transfer Kit, Invitrogen), before blocking with 1% (v/v) Western Blocking Reagent (WBR, Roche, Basel, Switzerland) in PBS for 30 min at RT or o/n at 4°C. Membranes were then incubated with 1 µg/mL biotinylated mAb anti-FHR-5.4 (to detect FHR-5) and anti-FHR-2.1 (to detect FHR-1 and FHR-2) for 1 h at RT. Membranes were washed three times in PT, followed by incubation for 1 h with 0.1% (v/v) strep-HRP and 0.5% (v/v) WBR in PBS, before washing three times in PT and two times in PBS. Membranes were developed with Pierce ECL 2 Western Blotting substrate kit (Pierce/Thermofisher Scientific) according to manufacturer’s instructions and visualized on a ChemiDoc MP System (Bio-Rad, Hercules, CA, USA). Images were analyzed using ImageLab (version 5.0; Bio-Rad).

### Sucrose Gradients

Sucrose gradients were used to separate FHR proteins based on size and density. Pooled sera of *CFHR1*-sufficient and -deficient donors (150 µL, diluted 1:2 in PBS) were loaded on 5–32.9% (w/v) sucrose (Merck, 1.07654) gradients in PBS, which had been generated using a pump system. Gradients were centrifuged for 20 h at 36,000 rpm (160,000*g*) using a SW 41 rotor (Beckman Coulter, Woerden, the Netherlands) after which they were fractionated in 24 fractions of 500 µL. All fractions were analyzed for presence of IgM, IgG, and albumin, using in-house ELISAs. FHRs were immunoprecipitated using 400 µL of each fraction, 50 µL of 200 µg/mL anti-FHR-5.5, and 500 µL RM-19-coupled sepharose, as described above.

### Conjugation of mAb Anti-FHR-2 with FLAG Peptide

Anti-FHR-2 was conjugated with FLAG peptide (Dris El Atmioui, Netherlands Cancer Institute, Amsterdam, the Netherlands), *via* a sulfo-SMCC hetero-crosslinker. Sulfo-SMCC was diluted to 10 mg/mL in deionized water and added 1:50 to 1 mg/mL anti-FHR-2. After 30 min at RT and dialysis to PBS o/n at 4°C, FLAG peptide with an additional C-terminal cysteine (DYKDDDDKC) was added at 200 µg/mL to anti-FHR-2 and incubated for 30 min at RT, followed by dialysis to PBS.

### ELISAs

All ELISAs were performed on Nunc Maxisorp 96-wells microtiter plates (Invitrogen), each step in a final volume of 100 µL at RT. Between incubation steps, plates were washed five times with PBS 0.02% (w/v) Tween-20, using a Biotek 405 LSRS (Biotek Instruments, Winooski, VT, USA). Assays were developed by addition of 100 µg/mL 3,5,3′,5′-Tetramethylbenzidine in 0.11 M sodium acetate containing 0.003% (v/v) H_2_O_2_, pH 5.5, and stopped by addition of 100 µL 2 M H_2_SO_4_. Absorbance was measured at 450 nm and corrected for absorbance at 540 nm using a Synergy 2 Multi-Mode plate reader (BioTek Instruments).

#### Cross-Reactivity and Competition ELISAs

To test cross-reactivity of all mAbs used in this study, 3 µg/mL RM-19 was coated o/n in PBS. Next, 1 µg/mL purified anti-FHR or anti-FH mAb was diluted in HPE and incubated on the plate for 1 h. Plates were then incubated for 1 h with 10 nM biotinylated rFHRs, or FH, diluted in HPE, followed by an incubation step of 25 min with strep-HRP, in PT. For competition experiments, 2 µg/mL purified anti-FHR or anti-FH mAb was used; rFHRs or FH were pre-incubated with 10 µg/mL possibly competing antibodies for 15 min, before addition to the plate.

#### FHR-1 and FHR-2 ELISAs for Homo- and Heterodimers

To measure FHR-1/1 homodimers in serum, 2 µg/mL aFH.02 was coated o/n in 0.11 M sodium acetate buffer, pH 5.5. Samples were diluted in HPE and incubated on the plate for 1 h. Subsequently, plates were incubated for 1 h with biotinylated anti-FH.02 (0.5 µg/mL in HPE). Plates were then incubated for 25 min with strep-poly-HRP and developed as described above. FHR-1/2 heterodimers in serum were tested by ELISA similar to the FHR-1/1 homodimer ELISA, except for the detection step in which biotinylated anti-FHR-2, diluted to 0.2 µg/mL in HPE, was used. To measure FHR-2/2 homodimers, plates were coated with 3 µg/mL anti-FLAG mAb o/n in PBS. After washing, plates were incubated for 1 h with anti-FHR-2^FLAG^ (0.5 µg/mL in HPE). Subsequently, plates were incubated with samples, diluted in HPE, for 1 h. Plates were then incubated for 1 h with biotinylated anti-FHR-2, before incubation with strep-poly-HRP and developed as described above.

#### FHR-5/5 Homodimer ELISA

To measure FHR-5/5 homodimers, 1 µg/mL anti-FHR-5.1 was coated o/n in 0.1 M sodium carbonate buffer, pH 9.6. Samples were diluted in HPE and incubated on the plate for 1 h. Subsequently, biotinylated anti-FHR-5.4 was added at 0.5 µg/mL in HPE for 1 h. Plates were then incubated with strep-poly-HRP and developed as described above.

### Ion Torrent Sequencing

Sequencing was performed as previously described (37). Briefly, an Ampliseq custom panel (Thermo Fisher Scientific, Waltham, MA, USA) “Sanquin Complement Panel,” was used to sequence the coding regions of various complement factors, including the *CFH-CFHR* locus.

### Heparin Affinity Chromatography

Serum-derived proteins were analyzed for their heparin-binding affinity using 1 mL HP Heparin columns (GE Healthcare) and an ÄKTA Avant HPLC system (GE Healthcare). Briefly, the columns were equilibrated with 10 mM NaPO_4_, before loading 150 µL serum that was diluted in 3 mL of 10 mM NaPO_4_, pH 7.4. Following washing, bound proteins were eluted using a linear salt gradient of 20 mL from 0 to 2 M NaCl. Fractions of 250 µL were collected in a deep 96-well plate, and analyzed by ELISA (26).

### Statistics

GraphPad Prism software versions 6.04 and 7.02 were used to analyze data and perform statistics (GraphPad Software, La Jolla, CA, USA). Significant differences were assessed by Mann–Whitney or Kruskal–Wallis tests. Correlations were assessed using a nonparametric Spearman’s correlation test.

## Results

### Dimers Present in Serum Do Not Include Heterodimers That Contain FHR-5

**Figure 1:**
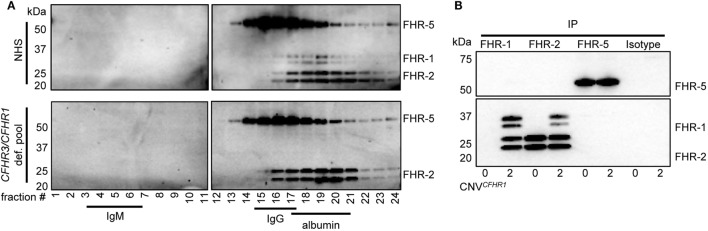
Serum-derived factor H-related (FHR)-1, FHR-2, and FHR-5 are present in fractions where dimers are expected in sucrose gradient. **(A)** Pooled sera were fractionated on a sucrose gradient, separating proteins on size and density and using IgM (750 kDa), IgG (150 kDa), and albumin (67 kDa) as reference proteins. FHR-1, FHR-2, and FHR-5 were precipitated from fractions using anti-FHR-5.5. Upper panel: normal human serum (NHS); lower panel: pooled *CFHR3/CFHR1*-deficient serum; visualized on Western blot with anti-FHR-2.1 (detecting FHR-1 and FHR-2) and anti-FHR-5.4 (detecting FHR-5). FHR-1 and FHR-2 circulate as two glycosylation variants, and thus are visualized as two distinct bands. Blots are representative of two pooled sera and four healthy donors. **(B)** Immunoprecipitation (IP) of FHR-1 (anti-FH.02), FHR-2 (anti-FHR-2), and FHR-5 (anti-FHR-5.1) from serum of healthy donors with zero or two gene copies of *CFHR1*, visualized on Western blot using anti-FHR-2.1 and anti-FHR-5.4. Blot is representative of *n* = 3. CNV, copy number variation.

To analyze the circulating molecular size of FHRs *in vivo* with minimal artifacts (e.g., without prior purification or enrichment steps), pooled sera from healthy individuals were fractionated on sucrose gradients. This allowed us to investigate the FHR composition as close as possible to the *in vivo* situation. IgM (900 kDa), IgG (150 kDa), and albumin (67 kDa) were utilized as protein size references. The presence of FHRs in the fractions was analyzed by immunoprecipitation (IP) and visualization on Western blot, using in-house generated monoclonal antibodies (Figure 1A).

When FHR-1 (37–42 kDa), FHR-2 (26–29 kDa), and FHR-5 (65 kDa) (Figure S1 in Supplementary Material) would circulate as dimers, the largest protein complex would be a FHR-5/5 homodimer (130 kDa), migrating similar to IgG. FHR-2/2 homodimers would form the smallest complex (52–58 kDa), migrating similar to albumin, closely followed by FHR-1/1 homodimers (74–84 kDa). Indeed, protein bands corresponding with FHR-5 were found in IgG-containing fractions, while FHR-1 and FHR-2 were found in albumin-containing fractions, demonstrating these proteins migrated predominantly as dimers and not as monomers. No high-intensity bands were found in fractions where higher-order oligomers or monomers would migrate, indicating that the majority (if not all) of FHR-1, FHR-2, and FHR-5 circulate *in vivo* as dimers. Although it was previously suggested that FHR-5 would form heterodimers with FHR-1 (11, 23), the highest intensity bands of FHR-5 did not shift in pooled *CFHR3/CFHR1*-deficient serum, suggesting that heterodimers containing FHR-1 and FHR-5 do not form.

As the resolution of the fractionation was too low to discriminate between homo- and heterodimers of FHR-1, FHR-2, and FHR-5, we aimed to verify FHR heterodimerization by IP from serum without prior purification steps. The highly specific anti-FHR-5.1 mAb only precipitated FHR-5 and did not co-precipitate FHR-1 or FHR-2 from healthy donor serum (Figure 1B). It is conceivable that FHR-2/FHR-5 might form in the absence of FHR-1, but also in donors carrying the *CFHR3/CFHR1* deletion, no FHR-2 was co-precipitated with anti-FHR-5.1. In contrast, FHR-1 and FHR-2 precipitated both with the FHR-1-specific anti-FH.02 and the FHR-2-specific anti-FHR-2, demonstrating FHR-1/2 heterodimers are present in serum. In line with our observations with anti-FHR-5.1, we did not detect any band corresponding to FHR-5 in the IP of anti-FH.02 or anti-FHR-2. No FHR-1/2 heterodimers were found in serum of donors carrying the *CFHR3/CFHR1* deletion.

### Recombinant FHR-1, FHR-2, and FHR-5 Exchange Monomers Rapidly

**Figure 2:**
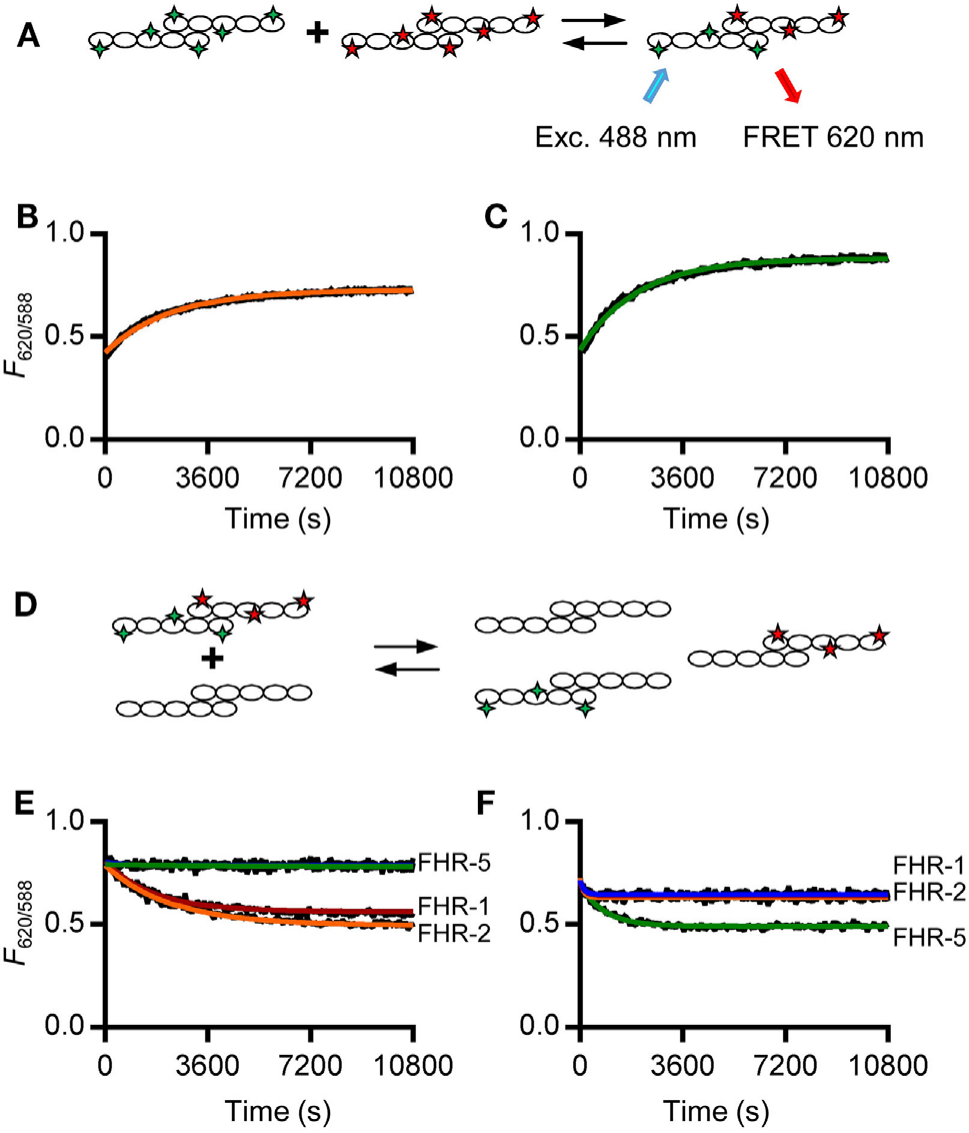
rFHR-1 and rFHR-2 form homo- and heterodimers *in vitro*, while rFHR-5 forms homodimers only. **(A)** Schematic overview of a monomer exchange experiment, to monitor reaction kinetics between fluorescently labeled recombinant human FHRs (rFHRs). Excitation of the 488-labeled monomer will lead to FRET toward the 594-labeled monomer, when in close proximity **(B)** rFHR-2^488^ and rFHR-2^594^ or **(C)** rFHR-5^488^ and rFHR-5^594^ were mixed and the exchange reaction was monitored in time. *Orange* and *green* lines represent fits of a first-order exponential. Graphs are representative of *n* = 5. **(D)** Schematic overview of a monomer exchange experiment, to monitor reaction kinetics between previously equilibrated fluorescently labeled rFHRs and a >5 times molar excess of native rFHRs. Previously equilibrated FHR-2^488/594^ **(E)** and FHR-5^488/594^ **(F)** were mixed with an excess of native rFHR-1 (*red*), rFHR-2 (*orange*), rFHR-5 (*green*), or buffer control (*blue*). Graphs are representative of *n* ≥ 3.

In order to study the kinetics of the dimerization of FHR-1, FHR-2, and FHR-5, we analyzed the monomer exchange between rFHRs using FRET, analogous to previous studies on non-covalent subunit exchange (34, 36). We labeled rFHR-2 and rFHR-5 with either DyLight^488^ or DyLight^594^. When these fluorochromes are in close proximity, exciting DyLight^488^ allows for FRET toward DyLight^594^, emitting light at 620 nm (Figure 2A). This allows monitoring of the monomer exchange process upon mixing 488- and 594-labeled rFHR-2, which will result in increasing amounts of a product containing both fluorescent labels until equilibrium is reached. Indeed, upon mixing FHR-2^488^ with FHR-2^594^, we detected the emergence of a FRET signal (Figure 2B). A similar observation was made when rFHR-5^488^ and rFHR-5^594^ were mixed (Figure 2C). Both processes followed first-order kinetics with comparable observed rate constants (*k* = 5.6 × 10^−4^ s^−1^ and 6.8 × 10^−4^ s^−1^ for FHR-2/2 and FHR-5/5, respectively). In other words, the exchange process is relatively fast, with a dissociation half-life of less than 30 min.

To further study which of these rFHRs could homo- or heterodimerize, we mixed previously exchanged rFHR-2^488^ and rFHR-2^594^ with an excess of unlabeled rFHR-1, rFHR-2, or rFHR-5 (Figures 2D,E). When either unlabeled rFHR-1 or rFHR-2 was added, the observed rate constants (*k* = 5.3 × 10^−4^ s^−1^, *k* = 4.0 × 10^−4^ s^−1^, respectively) were equal to the observed rate constant when rFHR-2^488^ and rFHR-2^594^ exchanged monomers (Figure 2B). This confirms that the monomer exchange process occurs quickly and is not affected by the presence of fluorescent labels. Furthermore, it shows that rFHR-2 is able to form homodimers with itself as well as heterodimers with rFHR-1. As expected, when rFHR-5 was added, the signal remained unchanged, showing that rFHR-2 and rFHR-5 do not form heterodimers. Concordantly, we mixed rFHR-5^488^ and rFHR-5^594^ with an excess of unlabeled rFHR-1, rFHR-2, or rFHR-5 (Figure 2F). When unlabeled rFHR-5 was added, the FRET signal decreased (with a rate constant of *k* = 1.2 × 10^−3^ s^−1^), confirming that the FRET signal increase seen previously (Figure 2C) was due to monomer exchange of rFHR-5. In line with our previous results with unlabeled FHR-5 not being able to decrease FHR-2 homodimerization (Figure 2E), addition of either unlabeled rFHR-1 or rFHR-2 did not decrease the FRET signal caused by FHR-5 homodimerization.

Together, these results, using both serum-derived and recombinant FHRs, show that FHR-1 and FHR-2 are equally able to form either homo- or heterodimers. In contrast to previous reports (11, 31), but in line with IP experiments shown here, we observed that rFHR-5 only forms homodimers. Our observations of both recombinant and serum-derived FHRs demonstrate that four dimers are present under physiological conditions: FHR-1/1, FHR-2/2, and FHR-5/5 homodimers, as well as heterodimers containing FHR-1 and FHR-2.

### FHR-1/1 Homodimers Are the Most Prevalent FHR Dimer in the Circulation

After determining their exact *in vivo* composition, we set out to determine the serum concentrations of FHR-1/1, FHR-2/2 and FHR-5/5 homodimers. As rFHR-1 and FHR-2 showed a rapid monomer exchange, we anticipated to find FHR-1/2 heterodimers as well.

Generation of monospecific mAbs toward serum-derived FHR-1 was unsuccessful, as all anti-FHR-1 mAbs obtained either cross-reacted with FH or one or more FHRs. Instead, to distinguish between FHR-1 and FH, we made use of antibodies that recognize epitopes in FHR-1 that, due to dimerization, would be present twice in FHR-1/1 homodimers, but only once in monomeric FH. We did not obtain mAbs directed against SCR4, which is identical to SCR19 of FH. Additionally, we did not use mAbs reactive toward SCR3 (95–100% identical to SCR18 of FH) to exclude the possibility that the measurements would be affected by the two different allotypes of FHR-1: FHR-1*A and FHR-1*B (33). Six mAbs were reactive toward SCR5 (97% identical to SCR20 of FH), but did not recognize the same epitope as indicated by competition ELISA. Five of these mAbs showed similar titrations of both normal human serum (NHS) and rFHR-1 (Figure S2 in Supplementary Material). No signal was observed when *CFHR3/CFHR1*-deficient serum was tested, confirming that FH was not detected and thus confirming the specificity of the FHR-1/1 homodimer assays. Those with parallel curves all showed a similar ratio between NHS and rFHR-1, suggesting that the measurement of FHR-1/1 homodimers was not affected by the specific epitope targeted. Taking into account sensitivity and specificity, we continued with anti-FH.02 (Figures S3 and S4A in Supplementary Material).

**Figure 3:**
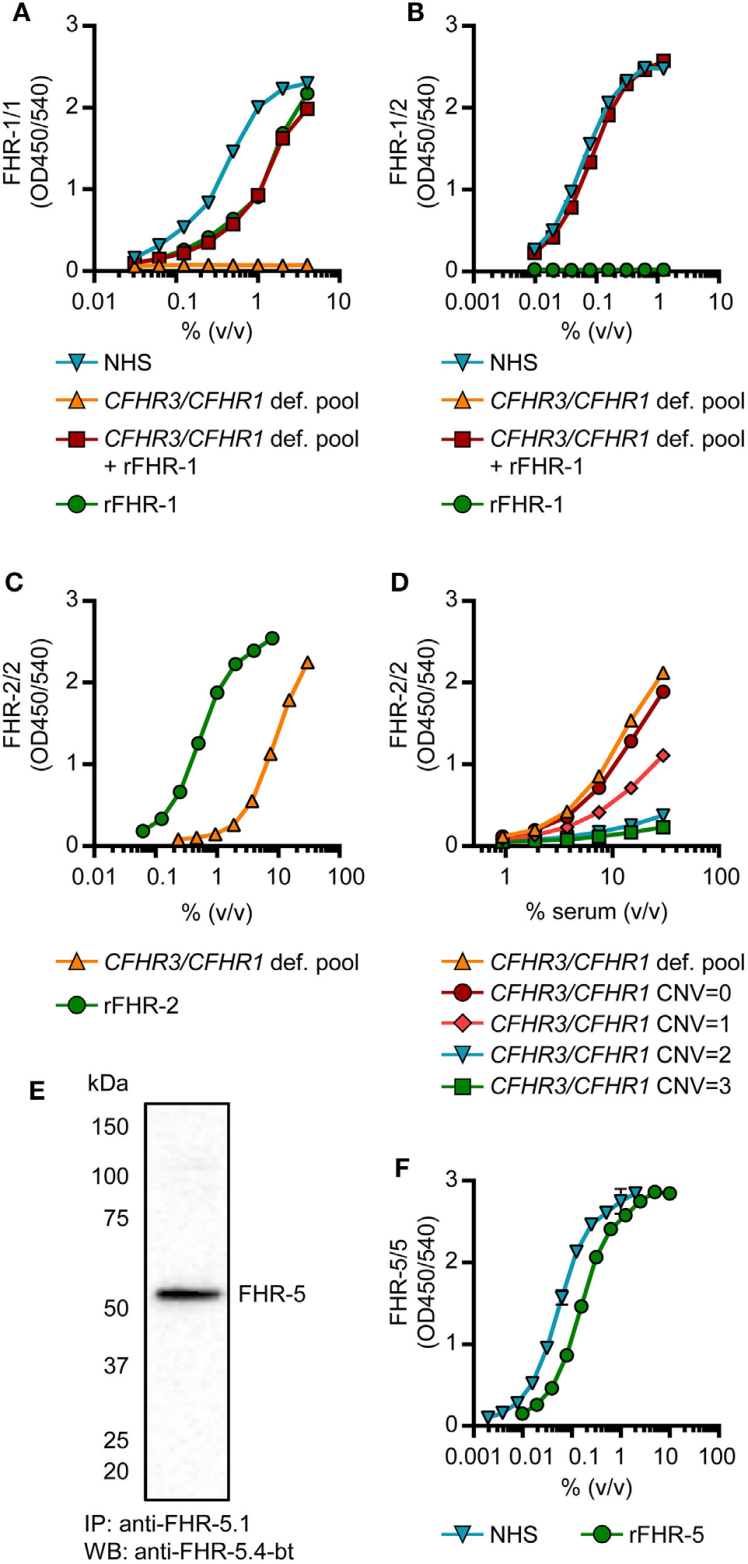
Development of factor H-related (FHR)-1/1, FHR-1/2, FHR-2/2 and FHR-5/5 ELISAs. **(A,B)** Titration of NHS, pooled *CFHR3/CFHR1*-deficient serum, pooled *CFHR3/CFHR1*-deficient serum spiked with 5 µg/mL rFHR-1 and 5 µg/mL rFHR-1 in the FHR-1/1 **(A)** and FHR-1/2 **(B)** ELISAs. Pooled *CFHR3/CFHR1-*deficient serum was incubated with 5 µg/mL rFHR-1 at 37°C for >2 h to allow for monomer exchange. **(C,D)** Titration of the pooled *CFHR3/CFHR1-*deficient serum and 50 µg/mL rFHR-2 in the FHR-2/2 ELISA **(C)** and in representative healthy donors with varying copy number variation (CNV) of *CFHR3/CFHR1* **(D)**. **(E)** Immunoprecipitation from NHS, using anti-FHR-5.1, visualized on Western blot using anti-FHR-5.4 **(F)** Titration of 0.5 µg/mL rFHR-5 and NHS in the FHR-5/5 ELISA. All graphs are representative of *n* = 3; points represent means with error bars indicating SD of duplicates. NHS, normal human serum.

The FHR-1/1 homodimer assay, using anti-FH.02 as capturing and detecting mAb, showed identical curves when rFHR-1 was spiked in *CFHR3/CFHR1*-deficient serum when compared to NHS (Figure 3A). The protein concentration of rFHR-1 was determined with an extinction coefficient of 1.74 (280 nm, 0.1% (w/v) solution, incl. 6× His-tag) to calibrate NHS, which was determined to contain 11.5 µg/mL FHR-1/1 homodimers (146 nM, based on MW of 79 kDa). As this concentration of FHR-1 was lower than suggested by recent reports (29, 30), we checked the relative concentration of FHR-1 in comparison to FH on Western blot using our characterized mAbs, and found that FHR-1 circulates in serum at an average 1:10 ratio compared to FH, in line with our ELISA results (Figure S5 in Supplementary Material).

Factor H-related-1/2 heterodimers were measured using anti-FH.02 as capturing mAb, and the monospecific anti-FHR-2 as detecting mAb (Figure 3B; Figure S3 and S4B in Supplementary Material). Similar to the FHR-1/1 assay, specificity was confirmed using *CFHR3/CFHR1*-deficient serum.

A FHR-2/2 homodimer ELISA was developed using the monospecific anti-FHR-2 that served both as a capturing and detecting antibody (Figure S4C in Supplementary Material). The capturing anti-FHR-2 was conjugated with a FLAG-tag, to be bound by an anti-FLAG antibody, to improve the sensitivity of the assay. The *CFHR3/CFHR1*-deficient serum titration ran parallel to rFHR-2, while the presence of FHR-1 in *CFHR3/CFHR1*-sufficient serum interfered with the detection of FHR-2/2 homodimers; the more genes, the more interference, thus suggesting a link between genotype and levels (Figures 3C,D). The pooled *CFHR3/CFHR1*-deficient serum was determined to contain 3.1 µg/mL FHR-2/2 homodimers (56 nM, based on MW of 55 kDa), using rFHR-2 as a reference with an extinction coefficient of 1.62 (280 nm, 0.1% (w/v) solution, incl. 6× His-tag). Due to formation of FHR-1/2 heterodimers in most sera, it was anticipated that FHR-1/2 heterodimers were interfering with the detection of FHR-2/2 homodimers.

Assuming that (1) FHR-1 and FHR-2 randomly associate in serum, (2) all the molecules are dimeric, and (3) the monomer exchange is a quick process, we calculated the concentration of FHR-1/2 heterodimers in NHS to be 5.2 µg/mL (78 nM, based on MW of 67 kDa), using the spike of rFHR-1 in *CFHR3/CFHR1*-deficient serum as standard curve. Using distribution laws [e.g., in classic genetics, and as described previously for IgG4 half-molecules (38)], we then inferred levels of FHR-2/2 homodimers in NHS, determining the FHR-2/2 homodimer concentration in NHS at 0.5 µg/mL (11 nM).

We obtained two monospecific mAbs against FHR-5, and used these to measure FHR-5/5 homodimers in serum (Figures S3 and S4D in Supplementary Material). The specificity of the assay was confirmed by Western blotting, as no FHR-5-deficient serum was available (Figures 3E,F). The protein concentration of rFHR-5 was determined using an extinction coefficient of 1.49 (0.1% (w/v) solution, 280 nm, incl. 6×His-tag), setting NHS at 1.49 µg/mL (11 nM, based on MW of 130 kDa).

### Functional Difference of FHRs in Binding to Heparin

Being able to measure all dimer species in a highly sensitive and specific manner, we determined each of their heparin-binding capacities, as surrogate to polyanionic residues on host cell surfaces. Although binding capacities were known for rFHR-5 (39), and suggested for serum-derived FHR-1 and FHR-2 (11), none had determined the exact capacity of each dimer species separately, without prior purification steps. We loaded serum (NHS, pooled *CFHR3/CFHR1*-deficient serum) directly onto heparin columns. Heparin-bound proteins were eluted by a linear increasing salt gradient and the FHRs were detected in all eluted fractions with our newly developed ELISAs.

**Figure 4:**
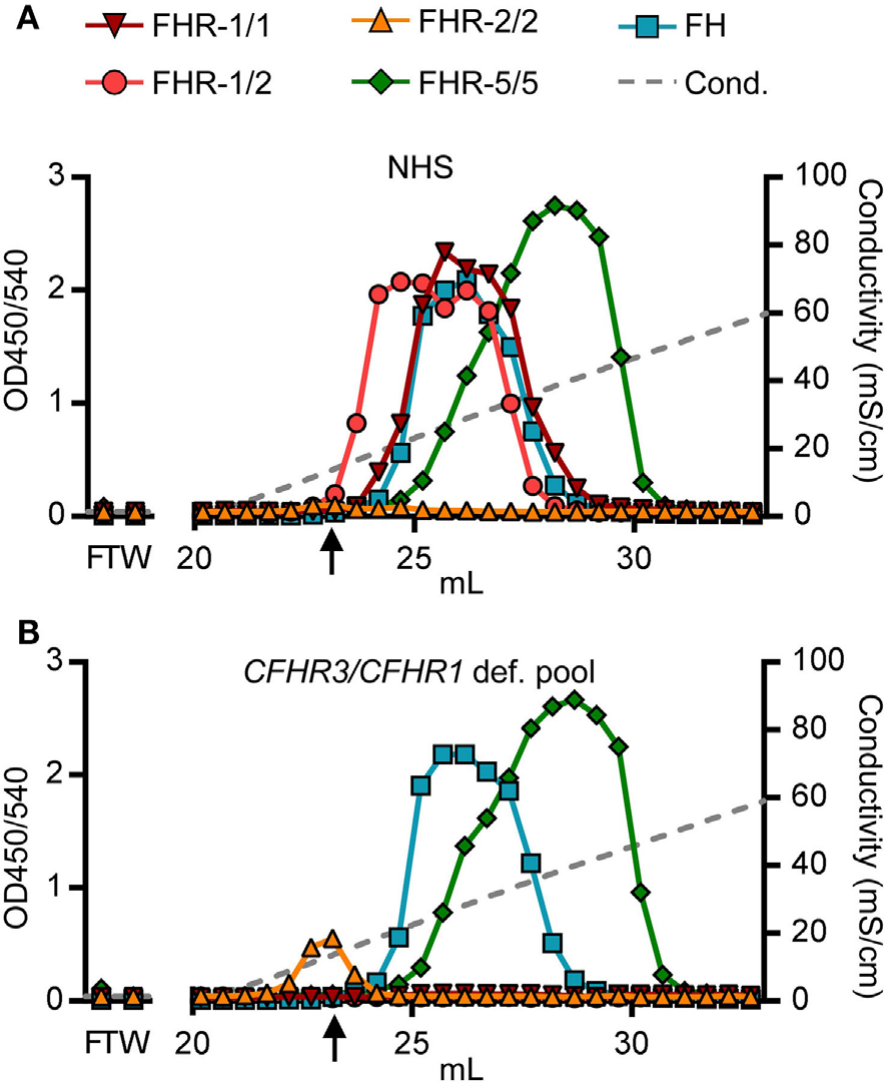
Heparin elution patterns using untreated sera. Fractions were analyzed by ELISA. **(A)** Heparin elution pattern of normal human serum (NHS) and **(B)** pooled *CFHR3/CFHR1*-deficient serum. Arrow indicates a conductivity of 14.06, representing the physiological salt concentration. Graphs are representative of *n* = 3. FT, flow through; W, wash.

As suggested for rFHR-5, serum-derived FHR-5/5 homodimers eluted at a conductivity of ~19.0 mS/cm (Figure 4A). The elution of FHR-5/5 homodimers was not affected by presence of FHR-1, again confirming that FHR-5 does not form heterodimers with FHR-1 (Figure 4B). In accordance with Tortajada *et al*. (11), FHR-1/1 homodimers eluted at a conductivity of ~16.5 mS/cm, the same conductivity at which FH eluted from the heparin column. However, FHR-1 eluted at lower ionic strength when dimerized with FHR-2 (~11.5 mS/cm), while FHR-2/2 homodimers showed no adequate binding strength at all (8.8 mS/cm), and was unable to bind heparin at physiological salt conditions (~14.06 mS/cm). While these results confirm the expected differences in binding capacities of each FHR protein, we now show that also the composition of plasma-derived FHR-1-containing dimers affects their binding capacity.

### Serum Levels of FHR-1, FHR-2, and FHR-5 in a Healthy Donor Cohort

Having shown that the composition of dimers determines ligand specificity, we determined the serum levels of each dimer species, making use of a healthy donor cohort, genotyped for *CFHR1* CNV by MLPA (Table S1 in Supplementary Material) (26).

**Figure 5:**
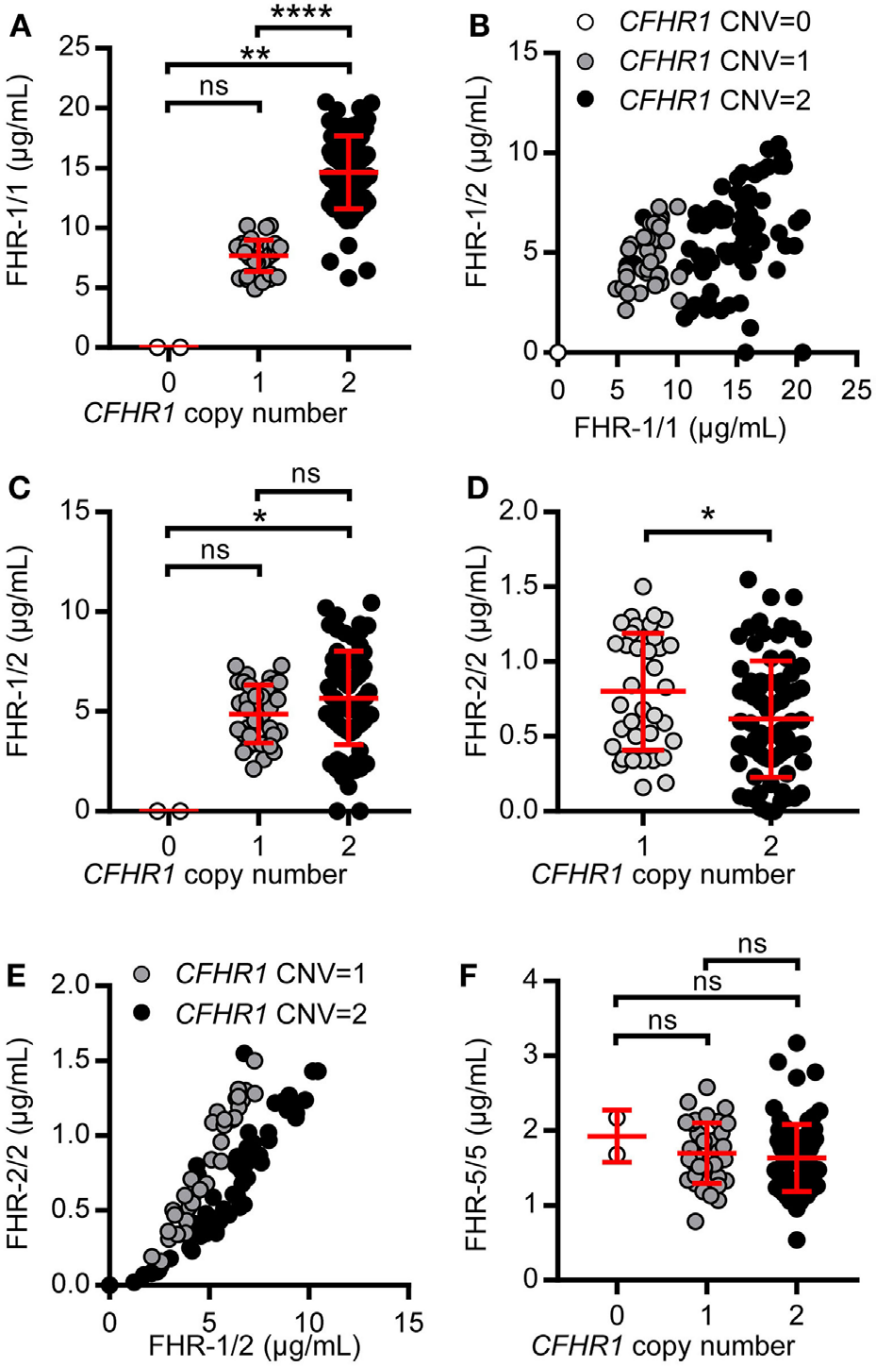
Levels of factor H-related (FHR)-1, FHR-2, and FHR-5 in a healthy donor cohort. A healthy donor cohort (*n* = 115) was measured for serum levels of FHR-1/1 **(A)**, FHR-1/2 **(C)**, FHR-2/2 **(D)**, and FHR-5/5 **(F)**. Donors are separated based on *CFHR1* gene copies, as determined by multiplex ligation-dependent probe amplification (MLPA). **(B,E)** Depicting the relation between FHR-1/1 and FHR-1/2 levels or FHR-1/2 and FHR-2/2, respectively. **(E)** Donors who carried no copies of *CFHR1* were not included in the graph. Data were analyzed using the Kruskal–Wallis test with Dunn’s test to correct for multiple comparisons **(A,C,F)**, Spearman’s test for correlation **(B, E)** and a Mann–Whitney test **(D)**.

Serum levels of FHR-1 were strongly determined by the CNV in *CFHR1* (Figure 5A). Individuals carrying two gene copies of *CFHR1* had more FHR-1/1 homodimers (mean = 14.64 µg/mL, SD = 3.04 µg/mL) when compared to those with one copy of *CFHR1* (mean = 4.88 µg/mL, SD = 1.33 µg/mL), while FHR-1/1 homodimers were undetectable in donors homozygous for the *CFHR1* deletion. Although levels of FHR-1/1 homodimers correlated with levels of FHR-1/2 heterodimers [Figure 5B, *r*_s_ = 0.42, *p* = 0.01 (CNV*^CFHR1^* = 1); *r*_s_ = 0.39, *p* = 0.0004 (CNV*^CFHR1^* = 2)], FHR-1/2 heterodimer levels were not significantly affected by the CNV of *CFHR1* [Figure 5C, mean = 5.01 µg/mL, SD = 1.49 µg/mL (CNV*^CFHR1^* = 1); mean = 5.84 µg/mL, SD = 2.41 µg/mL (CNV*^CFHR1^* = 2)]. Nonetheless, inferred data showed FHR-2/2 homodimer levels to be significantly affected by the CNV of *CFHR1* [Figure 5D, *p* = 0.03; mean = 0.85 µg/mL, SD = 0.41 µg/mL (CNV*^CFHR1^* = 1); mean = 0.65 µg/mL, SD = 0.41 µg/mL (CNV*^CFHR1^* = 2)], and to be strongly correlated with FHR-1/2 heterodimer levels [Figure 5E, *r*_s_ = 0.96, *p* < 0.0001 (CNV*^CFHR1^* = 1), *r*_s_ = 0.94, *p* < 0.0001 (CNV*^CFHR1^* = 2)], demonstrating that FHR-1/2 levels are mostly determined by FHR-2 serum levels. As expected, FHR-5/5 homodimer levels were not affected by the presence of FHR-1 levels (Figure 5F, *p* = 0.3058, mean = 1.66 µg/mL, SD = 0.43 µg/mL).

**Figure 6:**
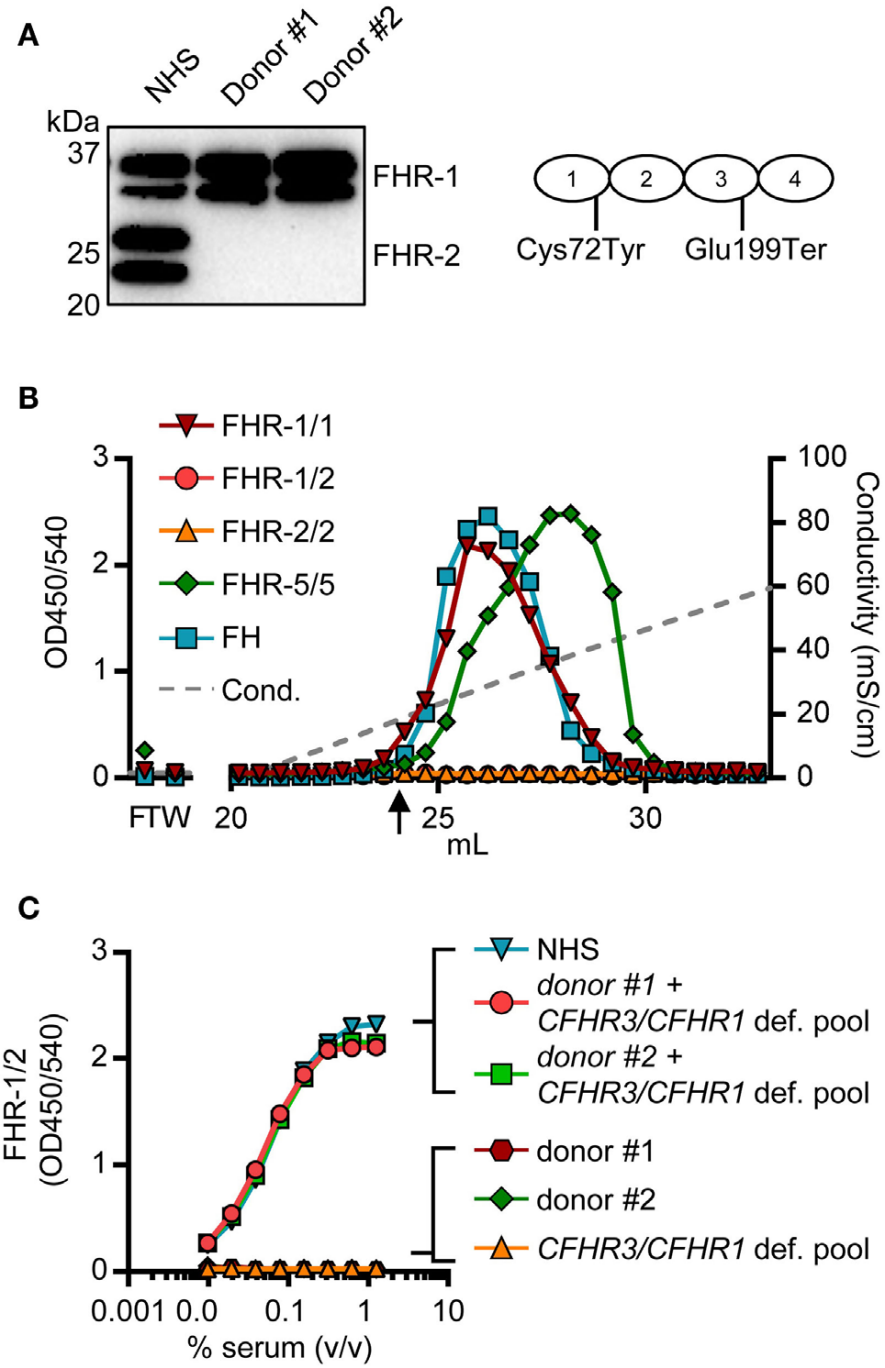
Two healthy donors lack factor H-related (FHR)-2 protein. **(A)** Lack of FHR-2 protein in two healthy donors as confirmed by immunoprecipitation using anti-FHR-2, visualized on Western blot by anti-FHR-2.1. Schematic representation of FHR-2 indicates mutations found by sequencing **(B)** Heparin elution pattern of *CFHR2*^mut/mut^ donor. Arrow indicates a conductivity of 14.06 mS/cm, representing the physiological salt concentration. **(C)** Titration of mixed sera in the FHR-1/2 ELISA. Equal amounts of serum were mixed and incubated at 37°C for >2 h to allow for monomer exchange. NHS, normal human serum.

Two donors that had two copies of both *CFHR1* and *CFHR2*, according to the MLPA results, showed a signal in the FHR-1/1 homodimer assay, but not in the FHR-1/2 heterodimer assay. The absence of FHR-1/2 heterodimers was confirmed by IP, demonstrating that these two individuals have no FHR-2 in serum (Figure 6A) as FHR-2 bands were completely lacking. Using a next generation sequencing platform for targeted analysis of complement factors (Ion Torrent; Sanquin Complement Panel; de Boer *et al*., manuscript in preparation; Table S2 in Supplementary Material), sequencing revealed two heterozygous mutations in the *CFHR2* gene of the two donors, c.215G >A (p.Cys72Tyr) and c.595G >T (p.Glu199Ter; *CFHR2*^mut/mut^; Figure S6 in Supplementary Material). FHR-1 and FHR-5 showed normal elution patterns when serum of one *CFHR2*^mut/mut^ donor was loaded onto heparin columns (Figure 6B). Having sera available that lacked either FHR-1 or FHR-2, allowed us to test whether FHR-1/2 heterodimers would also form *in vivo*. We mixed both sera and measured the appearance of FHR-1/2 heterodimers (Figure 6C). Indeed, similar to the quantity found in NHS, FHR-1/2 heterodimers were formed, indicating that the ability of heterodimerization with FHR-1 was not affected in these *CFHR2*^mut/mut^ donors. Furthermore, these data confirm our FRET data, showing that FHR-1 and FHR-2 can indeed exchange rapidly *in vivo*.

## Discussion

Human FHR-1, FHR-2, and FHR-5 have highly similar surface-binding domains as FH, the major complement-regulating protein in the circulation. Hence, it is hypothesized that they are able to compete with FH, and thereby fine-tune complement regulation. To date, accurate quantification of the three FH-related proteins in circulation was so far largely lacking or based on imprecise estimates (19, 29, 31, 32). Considering that FHR-1, FHR-2, and FHR-5 are thought to circulate in blood as homo- and heterodimers, accurate interpretation of these ELISAs is difficult (11, 23–25, 29, 30). In this study, we have now assessed the exact nature of the dimer composition and the serum levels of FHR-1/1, FHR-1/2, FHR-2/2, and FHR-5/5 dimers, being the only dimers that can form and circulate in blood.

Dimerization of FHR-1, FHR-2, and FHR-5 was demonstrated by IPs from sucrose gradient fractions and directly from serum. We did not detect monomers of the three proteins in serum. Oligomers were absent as well. Instead, we demonstrated that FHR-1, FHR-2, and FHR-5 are predominantly, if not completely, present in serum as homodimers, and that serum-derived FHR-1 and FHR-2 can form heterodimers as well. In contrast to previous reports (11, 23), we revealed a lack of FHR-5-containing heterodimers *in vivo*. Taken together, four dimers can be present *in vivo*: homodimers of FHR-1, FHR-2, and FHR-5, and heterodimers containing FHR-1 and FHR-2.

Using FRET, we showed that the monomer exchange of recombinant FHRs is a rapid process. This was confirmed by the formation of FHR-1/2 heterodimers when adding rFHR-1 to *CFHR1*-deficient serum. Also when mixing *CFHR1*-deficient with *CFHR2*^mut/mut^ serum, FHR-1/2 heterodimers were formed at levels comparable to those found in NHS. The kinetics suggests that FHR proteins might be excreted independently as homodimers and equilibrate as heterodimers in plasma—very rapidly and efficiently.

Three factors are essential for the presumed role that FHR-1, FHR-2, and FHR-5 play in complement activation and regulation: concentration, affinity for the target ligand and the avidity, which can be increased by dimerization. In this report, we showed for the first time accurate levels of each dimer species separately. We demonstrated that FHR-1/1 homodimers are the predominant FHR dimer present in serum. Nonetheless, all of the FHR dimers were present at concentrations at least an order of magnitude less than FH, which would make direct competition with FH less likely to occur *in vivo*. However, it is possible that FHR-1, FHR-2, and FHR-5 exert a direct function, regardless of FH, or that their concentrations would be higher in localized compartments.

Many reports have reported on the (relative) binding of FHR-1, FHR-2, and FHR-5 to deposited C3b, and all of these studies indicated affinities less than or at most comparable to FH (11, 23–25, 39–41). Next to C3b binding, association with polyanionic residues on cellular surfaces is essential for FH to exert its function (42, 43). FHR-5/5 homodimers, in concordance with previous reports (23, 39), eluted at a higher ionic strength from a heparin column than FH. As FHR-2/2 homodimers were unable to bind heparin at physiological salt conditions, it was not surprising that FHR-1/2 heterodimers eluted at a lower ionic strength than FHR-1/1 homodimers. These findings, together with the notion that the ratio between FHR-1/1 homodimers and FHR-1/2 heterodimers in NHS was only 1.8:1, indicate that each dimer should be measured separately and that the composition of each dimer is of influence on its ability to bind ligands.

We found much lower FHR-1/1 homodimer levels in Dutch healthy donors than recently reported for total FHR-1 levels (29, 30). However, in these particular publications, it was not clarified how their standard was quantified for FHR-1 levels. By using well-characterized mAbs, our assays specifically distinguish between FHR-1/1 homodimers and FHR-1/2 heterodimers, which is relevant as our results suggest that these may have different functional properties. Using both Western blot and ELISA, we found a ratio close to 1:10 for FHR-1 compared to FH, instead of 1:1.25 (29, 30), using seven different cross-reactive anti-FH and anti-FHR mAbs targeting the most C-terminal domain, which is neither involved in dimerization nor subject to allotype-specific variations (33).

Many reports have shown a link between genetics in the *CFHR* region and disease, demonstrating the functional consequences of having aberrant or no protein expression at all. Whether the levels of these dimers may change during acute disease because of liver dependent acute-phase reactivity or early clearance by binding to activated cells or debris, can now be addressed by direct protein measurements. This will shed light on the previously reported genetic associations of the *CFHR3/CFHR1* deletion with protection against AMD and increased susceptibility to aHUS, now that we can verify the protein levels of FHR-1/1 and FHR-1/2 dimers directly. Following CNV assessment by MLPA, our targeted NGS-based Complement Panel comprising 29 complement factors can be applied to verify aberrant protein measurements or MLPA results to identify novel genetic variants at the *CFH/CFHR* region, as was shown for the two donors who carry mutations in *CFHR2*. The two mutations occur at a minor allele frequency of 0.015 and 0.007, respectively, in the population (44). While the p.Cys72Tyr possibly leads to aberrant protein due to presence of free cysteines, similar to the loss of cysteine mutation as described for FH (2), the p.Glu199Ter mutation induces a free cysteine and a loss of SCR4, resulting in a lack of protein expression.

In conclusion, we have determined for the first time the exact composition of FHR dimers *in vivo* and demonstrated that FHR dimers could exchange quickly. We report the first accurate measurements of FHR-1/1, FHR-1/2, FHR-2/2, and FHR-5/5 concentrations in serum in a genotyped healthy donor cohort, using specific ELISAs for each type of dimer. FHR-1/1 homodimers were found to be the most dominant FHR dimer *in vivo*. As the FHR-2/2 serum levels are relatively low, FHR-2 is the limiting factor in formation of FHR-1/2 heterodimers. We did not find any evidence for the presence of FHR-1/5 or FHR-2/5 heterodimers in serum. The serum levels of FHR dimers were low, being at least at a 13-fold to 164-fold molar deficit compared to FH, suggestive that competition with FH in circulation is unlikely. Nonetheless, FHR-5 demonstrated higher binding strength toward heparin than FH. What the effect of this binding is on *in vivo* relevant surfaces remains to be elucidated. Functional studies focusing on FHR dimers must take the physiological levels and composition of these homo- and heterodimers into account.

## Ethics Statement

Blood samples were obtained from anonymous, healthy volunteers with informed, written consent in accordance with Dutch regulations, and this study was approved by the Sanquin Ethical Advisory Board in accordance with the Declaration of Helsinki.

## Author Contributions

AB, RP, DW, and TK designed research. AB, RP, MCB, GM, JG, PO, MB, and KL performed research. AB, RP, MB, KL, and TR analyzed data. AB, RP, TR, DW, and TK wrote the paper. All authors critically reviewed the manuscript, gave final approval of the version to be published, and agreed to be accountable for all aspects of the work in ensuring that questions related to the accuracy or integrity of any part of the work are appropriately investigated and resolved.

## Conflict of Interest Statement

The authors declare that the research was conducted in the absence of any commercial or financial relationships that could be construed as a potential conflict of interest.
