# Supplementary material for: Factor H-Related (FHR)-1 and FHR-2 Form Homo- and Heterodimers, while FHR-5 Circulates Only As Homodimer in Human Plasma
Source: Front Immunol. 2017 Oct 18;8:1328. doi: 10.3389/fimmu.2017.01328 (PMC5651247; doi:10.3389/fimmu.2017.01328)
Supplement: Supplementary file 1 [file Data_Sheet_1.docx]

Supplementary Material

**FHR-1 and FHR-2 form homo- and heterodimers, while FHR-5 circulates only as homodimer in human plasma**

**Anna E. van Beek^1,2,*^, Richard B. Pouw^1,2^, Mieke C. Brouwer^1^, Gerard van Mierlo^1^, Judy Geissler^3^, Pleuni Ooijevaar-de Heer^1^, Martin de Boer^3^, Karin van Leeuwen^3^, Theo Rispens^1^, Diana Wouters^1^, Taco W. Kuijpers^1,2^**

*** Correspondence:** Anna E. van Beek: a.vanbeek@sanquin.nl

# Figure 1


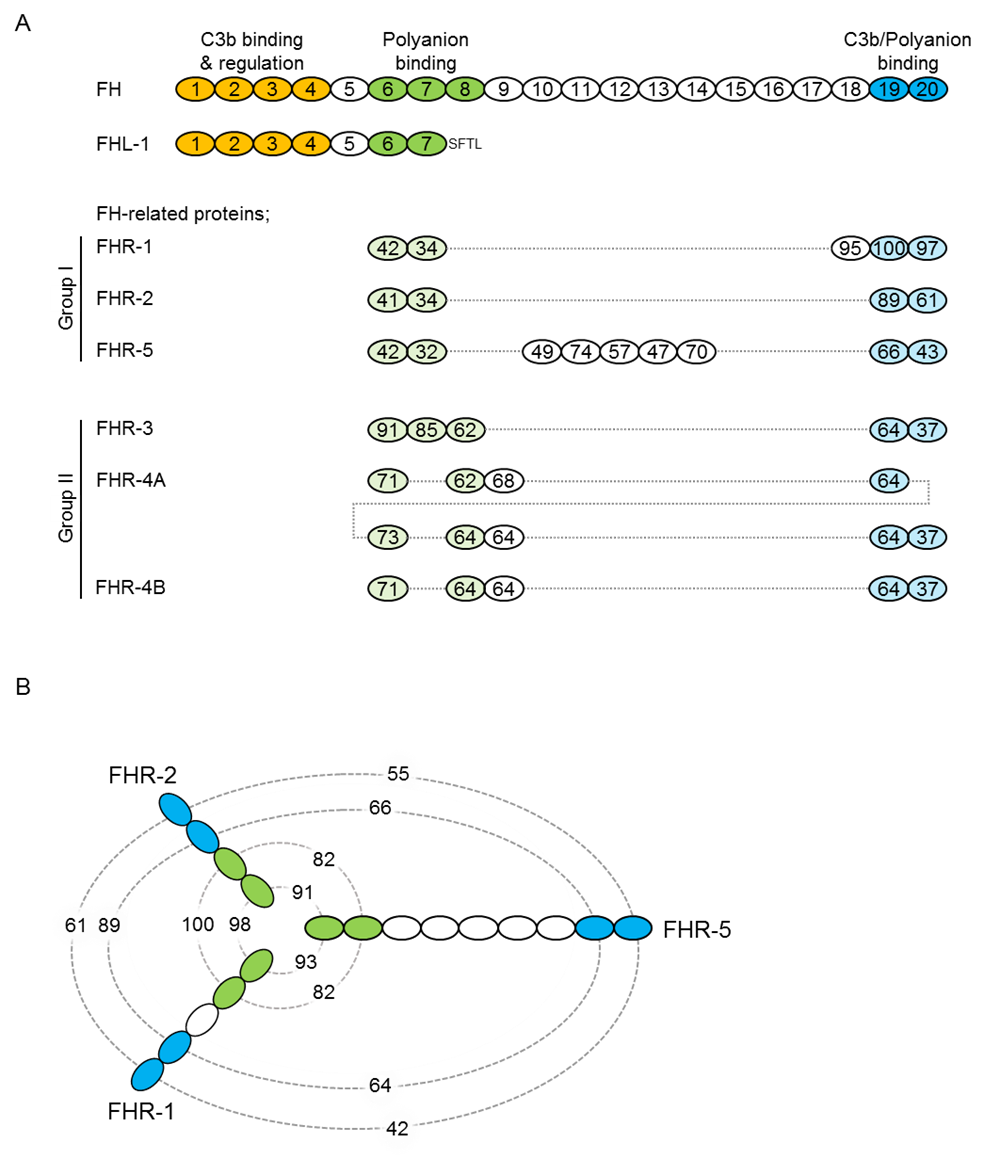


**Supplementary Figure 1.** Sequence similarity between FH and the FHRs. (*A*) Depicted are FH, the splice variant FHL-1 and the FHRs. Numbers indicate % amino acid sequence similarity compared to FH. FHRs are divided, based on similarity, into two groups. (*B*) The sequence similarity between FHR-1, FHR-2 and FHR-5. Dimerization motif is located within the two N-terminal domains indicated in green.

# Figure 2


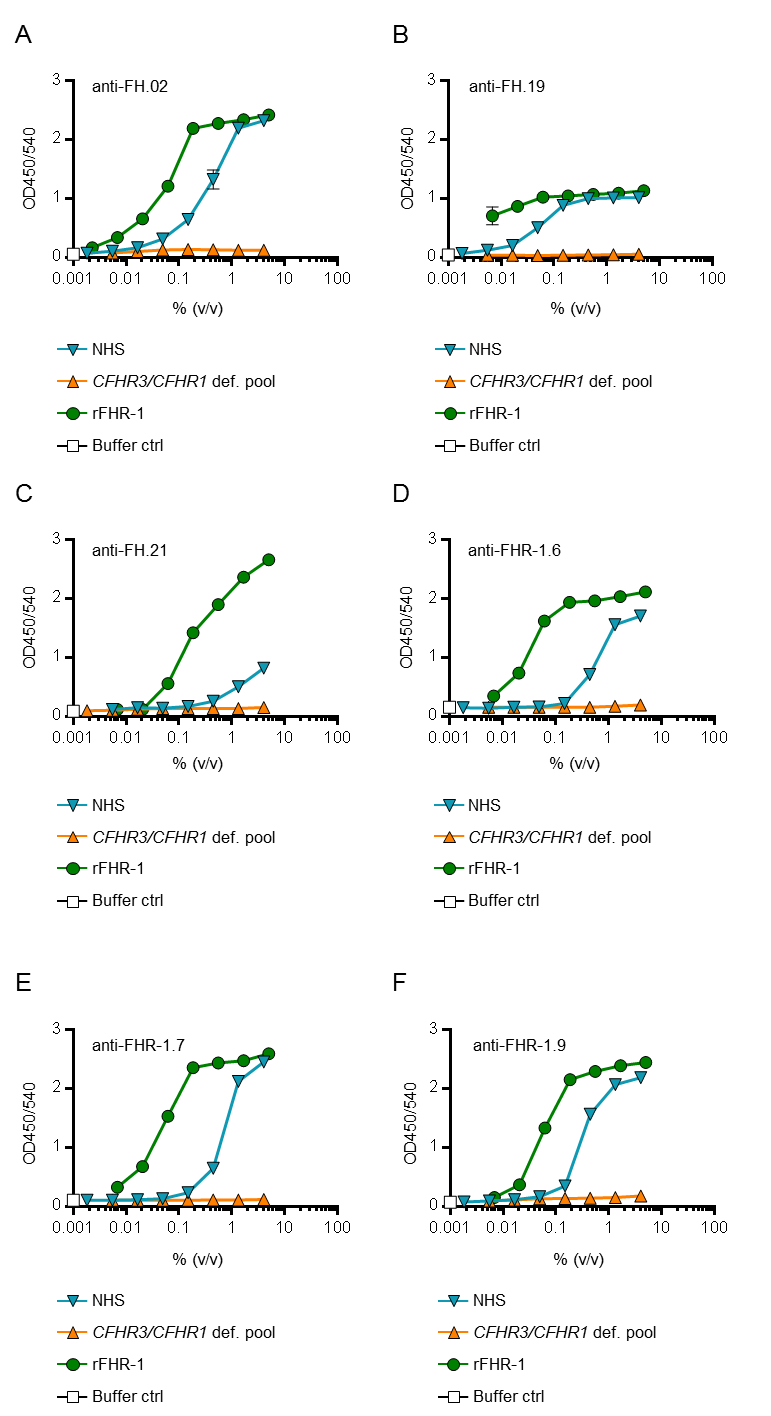


**Supplementary Figure 2.** FHR-1/1 homodimer sandwich ELISA set-ups, in which the same mAb is used both as a coating antibody and as a (biotinylated) detecting antibody. (*A*, *B*, *C*) ELISA set-ups using antibodies generated against FH, all targeting SCR20 of FH. Epitopes were mapped in a competition ELISA, using anti-FH antibodies that had been previously been epitope-mapped by fragments of FH. (*A*) set-up using anti-FH.02. (*B*) set-up using anti-FH.19. (*C*) set-up using anti-FH.21. (*D*, *E*, *F*) ELISA set-ups using antibodies generated against FHR-1, (*D*) using anti-FHR-1.6, (*E*) anti-FHR-1.7 and (*F*) anti-FHR-1.9.

# Figure 3

**Supplementary Figure 3.** Cross-reactivity of the anti-FH and anti-FHR mAbs used in this study. Wells were coated with indicated mAbs, followed by incubation with 10 nM of biotinylated rFHR or plasma-derived FH.

# Figure 4


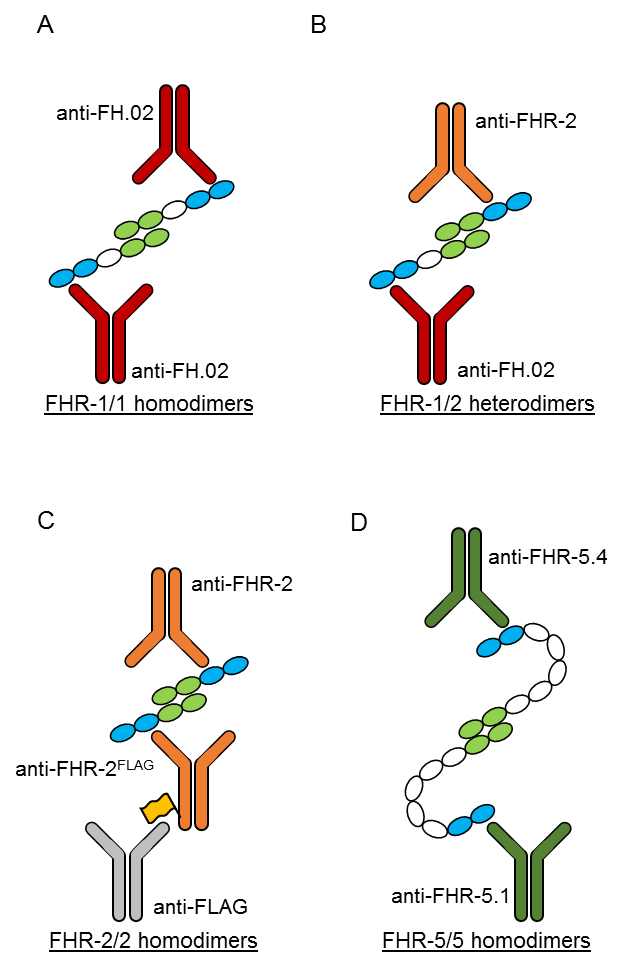


**Supplementary Figure 4.** Schematics of FHR dimer ELISAs as used in this study. (*A*) depicting ELISA to measure FHR-1/1 homodimers. (*B*) Depicting ELISA to measure FHR-1/2 heterodimers. (*C*) Depicting ELISA to measure FHR-2/2 homodimers. (*D*) Depicting ELISA to measure FHR-5/5 homodimers.

# Figure 5

**
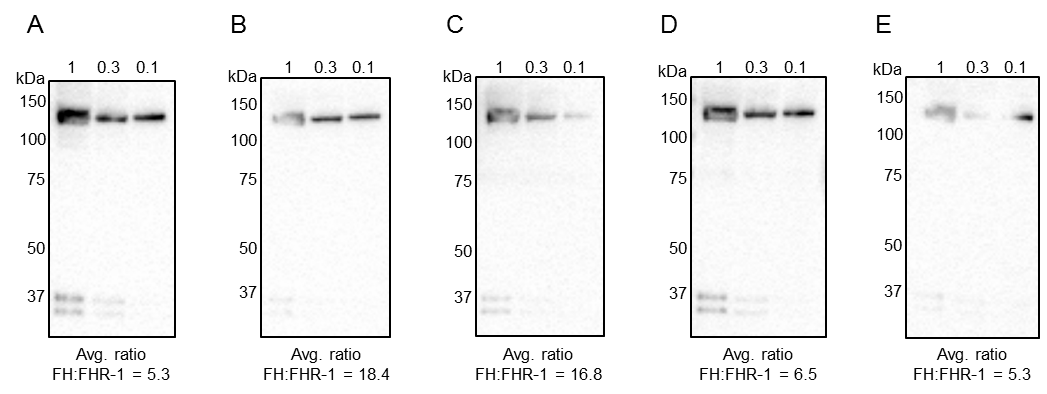
**

**Supplementary Figure 5.** Western blots showing the relative abundance of FHR-1 in comparison with FH. NHS was loaded directly onto SDS-page (starting at 1 µl, titrating 1:3) and visualized on Western blot using indicated mAbs and RM-19-HRP as a secondary mAb. Band intensities were analyzed using Image Lab 5.0. (*A*, *B*, *C*) Western blots using mAbs generated against rFHR-1, targeting SCR5: (*A*) using anti-FHR-1.9, (*B*) anti-FHR-1.7, or (*C*) anti-FHR-1.6. (*D*, *E*) Western blots using mAbs generated against FH, targeting SCR20: (*D*) using anti-FH.21, or (*E*) anti-FH.04.

# Figure 6


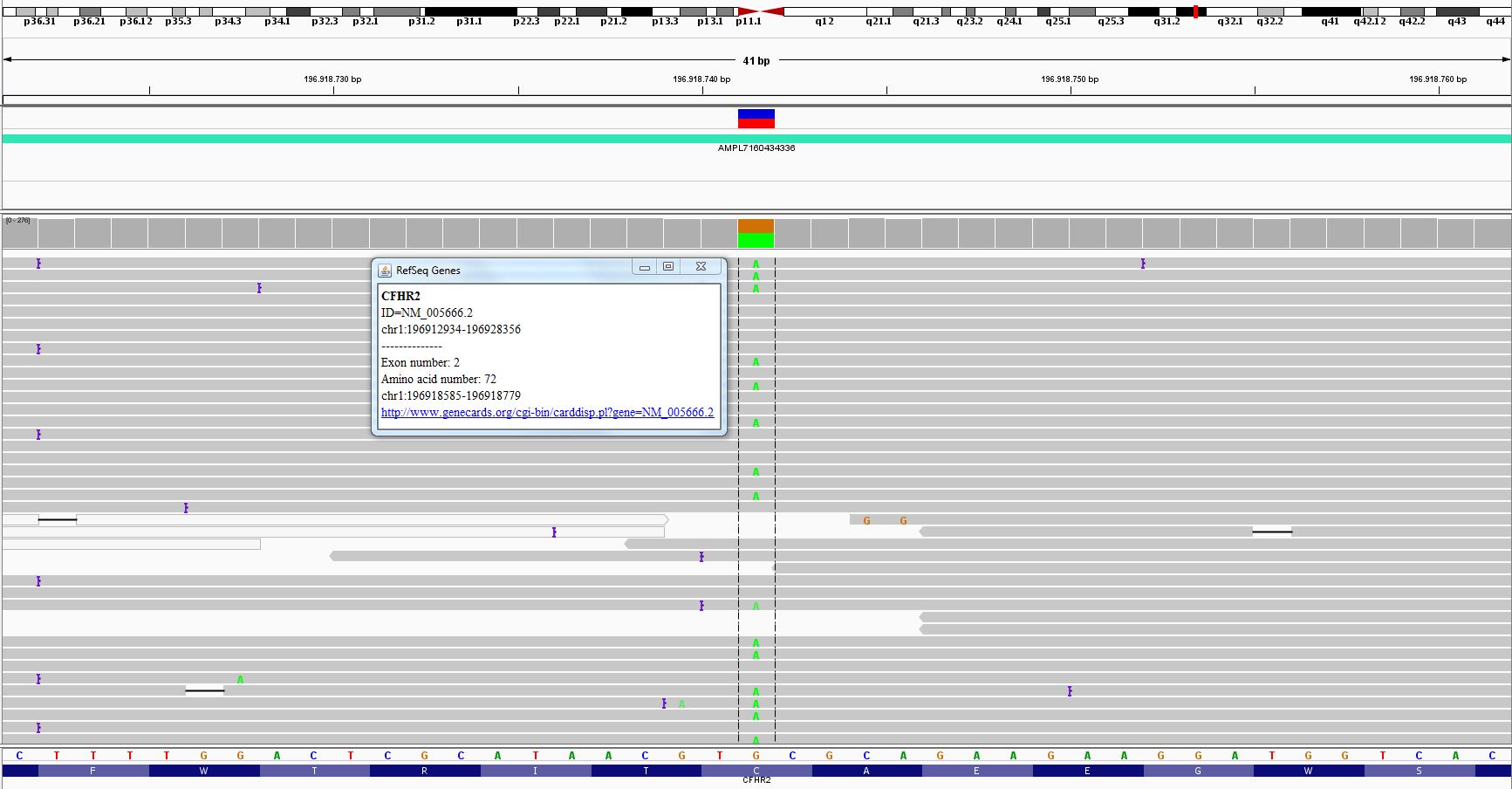


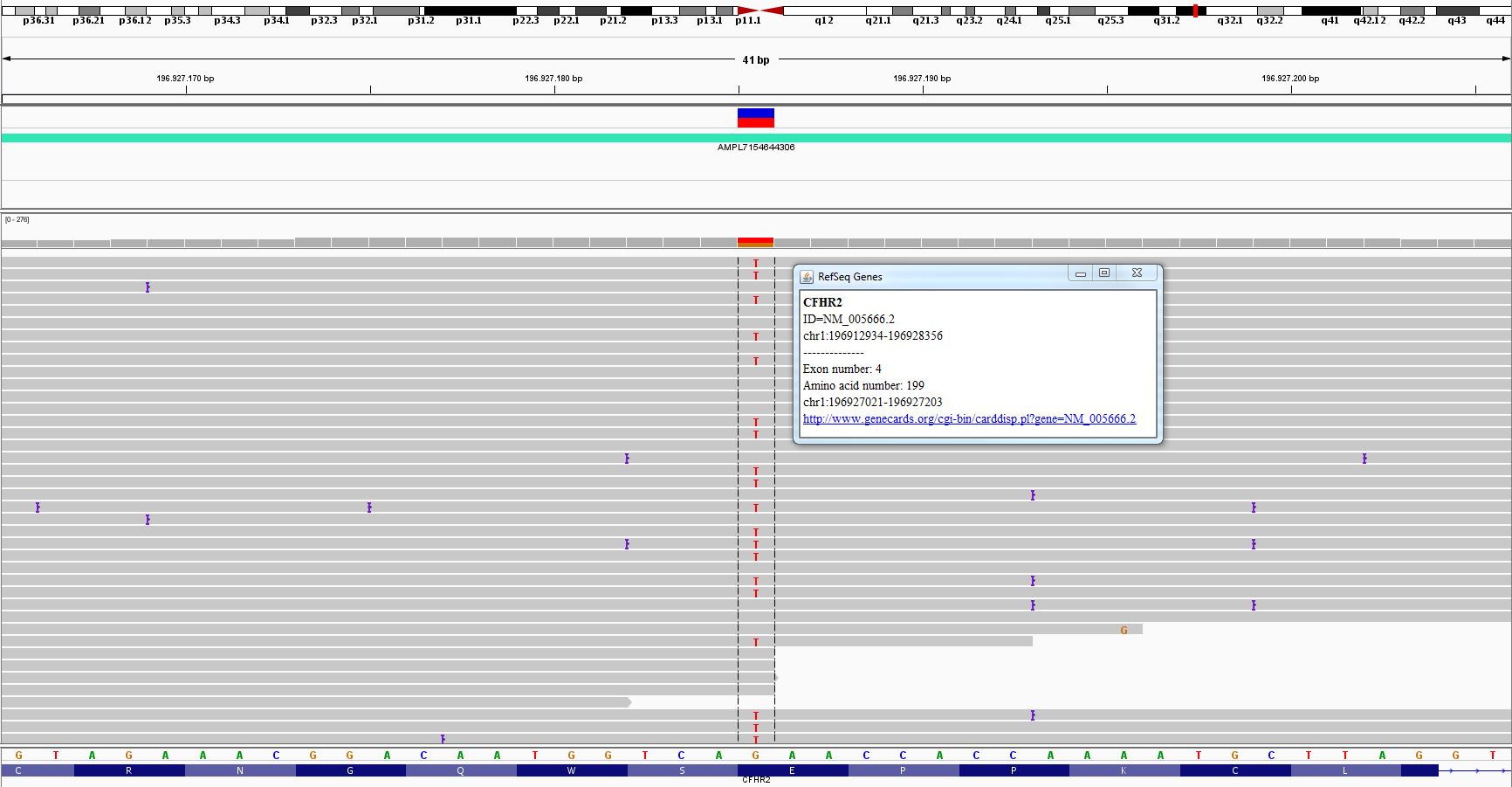


**Supplementary Figure 6**. Ion Torrent results of healthy donors who lack FHR-2. Both panels show the sequencing results of donor #1. The upper panel depicts the Cys72Tyr mutation, the lower panel depicts the Glu199Ter mutation.

# Supplementary Table 1. Copy number variation (CNV) analysis of the *CFHR3/CFHR1* deletion in the healthy donor cohort by Multiplex Ligation-dependent Probe Amplification (MLPA).

|  | ***CFHR3*** | ***CFHR1*** | **n** |
| --- | --- | --- | --- |
| *Gene copies* | 2 | 2 | 120 |
|  |  |  |  |
| *Deletion events* | 0 | 0 | 5 |
|  | 1 | 0 | 2 |
|  | 1 | 1 | 45 |
|  | 1 | 2 | 1 |
|  | 2 | 1 | 1 |
|  |  |  |  |
| *Duplication events* | 2 | 3 | 1 |
|  | 3 | 3 | 1 |
|  |  |  |  |
| **Total** |  |  | **176** |

# Supplementary Table 2. Sanquin Complement Panel as used on the Ion Torrent

| **Complement** | **Other** |
| --- | --- |
| *C3* | *THBD* |
| *C5* | *ADAMTS13* |
| *C6* | *DGKE* |
| *C7* | *MMACHC* |
| *C8A* | *PLG* |
| *C8B* |  |
| *C8G* |  |
| *C9* |  |
| *CD46* |  |
| *CD55* |  |
| *CD59* |  |
| *SERPING1* |  |
| *CFB* |  |
| *CFD* |  |
| *CFI* |  |
| *CFP* |  |
| *CFH* |  |
| *CFHR1* |  |
| *CFHR2* |  |
| *CFHR3* |  |
| *CFHR4* |  |
| *CFHR5* |  |
